# Polyadenosine diphosphate-ribose polymerase inhibitors: advances, implications, and challenges in tumor radiotherapy sensitization

**DOI:** 10.3389/fonc.2023.1295579

**Published:** 2023-12-04

**Authors:** Yi Zhang, Lijie Liang, Zheng Li, Ying Huang, Ming Jiang, Bingwen Zou, Yong Xu

**Affiliations:** ^1^ Department of Radiation Oncology, Division of Thoracic Oncology, Cancer Center, West China Hospital, Sichuan University, Chengdu, China; ^2^ Division of Head & Neck Tumor Multimodality Treatment, Cancer Center, West China Hospital, Sichuan University, Chengdu, China; ^3^ College of Management, Sichuan Agricultural University, Chengdu, China

**Keywords:** Polyadenosine diphosphate-ribose polymerase (PARP), cancer, radiation therapy, radiotherapy sensitization, PARP

## Abstract

Polyadenosine diphosphate-ribose polymerase (*PARP*) is a key modifying enzyme in cells, which participates in single-strand break repair and indirectly affects double-strand break repair. PARP inhibitors have shown great potential in oncotherapy by exploiting DNA damage repair pathways, and several small molecule PARP inhibitors have been approved by the U.S. Food and Drug Administration for treating various tumor types. PARP inhibitors not only have significant antitumor effects but also have some synergistic effects when combined with radiotherapy; therefore they have potential as radiation sensitizers. Here, we reviewed the advances and implications of PARP inhibitors in tumor radiotherapy sensitization. First, we summarized the multiple functions of PARP and the mechanisms by which its inhibitors exert antitumor effects. Next, we discuss the immunomodulatory effects of *PARP* and its inhibitors in tumors. Then, we described the theoretical basis of using PARP inhibitors in combination with radiotherapy and outlined their importance in oncological radiotherapy. Finally, we reviewed the current challenges in this field and elaborated on the future applications of PARP inhibitors as radiation sensitizers. A comprehensive understanding of the mechanism, optimal dosing, long-term safety, and identification of responsive biomarkers remain key challenges to integrating PARP inhibition into the radiotherapy management of cancer patients. Therefore, extensive research in these areas would facilitate the development of precision radiotherapy using PARP inhibitors to improve patient outcomes.

## 1 Introduction

Cancer poses a formidable challenge to the medical community despite extensive research and advancements in therapeutic strategies (1, 2). Radiotherapy remains a cornerstone in the management of various cancers. However, radioresistance and the potential of damage to the healthy tissues surrounding tumors necessitate the development of strategies that can enhance the therapeutic index of radiotherapy with minimal side effects. Notably, a class of drugs known as radiosensitizers has shown great potential in improving the therapeutic efficacy of radiotherapy because they enhance the susceptibility of cancer cells to the damaging effects of radiation. Poly (ADP-ribose) polymerase (*PARP*), which senses DNA damage and participates in repair, has emerged as a promising target for radiotherapy sensitization. The polymerase engages in the repair of single-strand breaks (SSBs) via the base excision repair (BER) pathway (3). Additionally, *PARP* is involved in non-homologous end joining (NHEJ) (4). In particular, Schultz et al. (5) indicates that while PARP1 may play a role in controlling DNA damage recognized by homologous recombination (HR), it does not seem to be directly involved in executing HR. PARP1 is the most abundant and active enzyme in the PARP family (6). It is triggered by the presence of damaged DNA and forms poly ADP-ribose (PAR) (6). Then, it simultaneously recruits the scaffold protein X-ray repair cross-complementing protein 1 (*XRCC1*) to the site of DNA damage, forming a complex with DNA ligase III and DNA polymerase β to participate in the SSB repair through the BER pathway (3). In 2005, Farmer et al. and Bryant et al. reported that the inherent dysfunction of HR in cells with BRCA1/2 mutations increases their sensitivity to PARP inhibitors (7, 8). PARP inhibitors act on single-strand DNA repair and induce the collapse of DNA replication forks. Consequently, SSBs are converted into more severe double-strand breaks (DSBs) (9), leading to cell apoptosis. The effect of PARP inhibitors is magnified in cancers exhibiting homologous recombination defects (HRD) through a mechanism known as synthetic lethality. The concept of radiosensitization by PARP inhibitors was identified early on, even before the synthetic lethality theory was validated, with clinical trials of radiotherapy in combination with PARP inhibitors conducted in response to this. To acknowledge the historical context, studies as early as the 1980s, such as the one by Ben-Hur et al. (10), used the prototype inhibitor 3AB and contributed to the understanding of this field. Today, this area of research has led to the licensing of six PARP inhibitors, reflecting the significant progress made since these initial investigations. These inhibitors have been approved for the treatment of breast and ovarian cancers with *BRCA1/2* mutations (11). Several PARP inhibitors are in clinical trial stages and not only bind with the active sites of *PARP1* but also closely associate with the active sites of *PARP2* and *PARP3*. This indicates that PARP inhibitors can exert antitumor effects by inhibiting multiple PARP enzymes (12). Moreover, *in vitro* and *in vivo* studies suggest that PARP inhibitors can be used as sensitizer drugs in conjunction with chemotherapy agents, such as temozolomide (13), in cancer treatment (14, 15). Given the pivotal role of *PARP* in DNA damage repair, many researchers have explored the potential of these inhibitors as radiosensitizers. The inhibition of PARP-mediated DNA damage repair in cells can sensitize them to radiation by prolonging DNA strand breaks and activating cell death signaling pathways with relatively low toxicity (16–19). Over the past decade, PARP inhibitors have been effectively used as radiosensitizers in the radiotherapy of various malignancies, including lung cancer (20), breast cancer (21), and gliomas (22). Lesueur et al. (23) conducted a systematic review of 64 studies and found that 61 of these studies reported the radiosensitizing effects of PARP inhibitors (23). The median sensitization enhancement ratio of PARP inhibitors ranged from 1.3 in prostate tumors to 1.5 in lung cancer, emphasizing the potential of PARP inhibitors as radiosensitizers (24).

Therefore, understanding the role and current status of PARP inhibitors in tumor radiotherapy sensitization is imperative for the development of more effective cancer treatment strategies. Here, we aimed to provide researchers, clinicians, and oncologists with an overview of the advances in understanding the role of PARP inhibitors for developing effective therapeutic strategies for cancer. We reviewed the current research on the role of PARP1 inhibitors in tumor radiotherapy sensitization. First, we described the structure and biological function of PARP1 and how its inhibition exerts antitumor and radiotherapy-sensitizing effects. Subsequently, we summarized the role of PARP inhibitors in the regulation of various types of immune cells. Then, we discussed the findings of completed and ongoing clinical trials of PARP inhibitors. Finally, we elaborated on the challenges of integrating these inhibitors into standard cancer treatment regimens.

## 2 Advances in PARP1 and its inhibitors

### 2.1 Structure and function of *PARP1*

PARP, a multifunctional post-translational modifying enzyme present in most eukaryotic cells, is extensively involved in DNA damage repair. The *PARP* family comprises 17 members, which exhibit varied cellular functions (25). *PARP1*, *PARP2*, and *PARP3* have crucial roles in cellular processes such as DNA repair, translation, cell signaling, and cell cycle regulation, primarily because of their capability to catalyze poly ADP ribosylation. On the other hand, tankyrase 1 (*PARP5a*) and tankyrase 2 (*PARP5b*) are significant for telomere maintenance (6). *PARP1* is the most extensively studied core member and undertakes most of the crucial functions of the *PARP* family.

#### 2.1.1 Structure of *PARP1*

**Figure 1:**
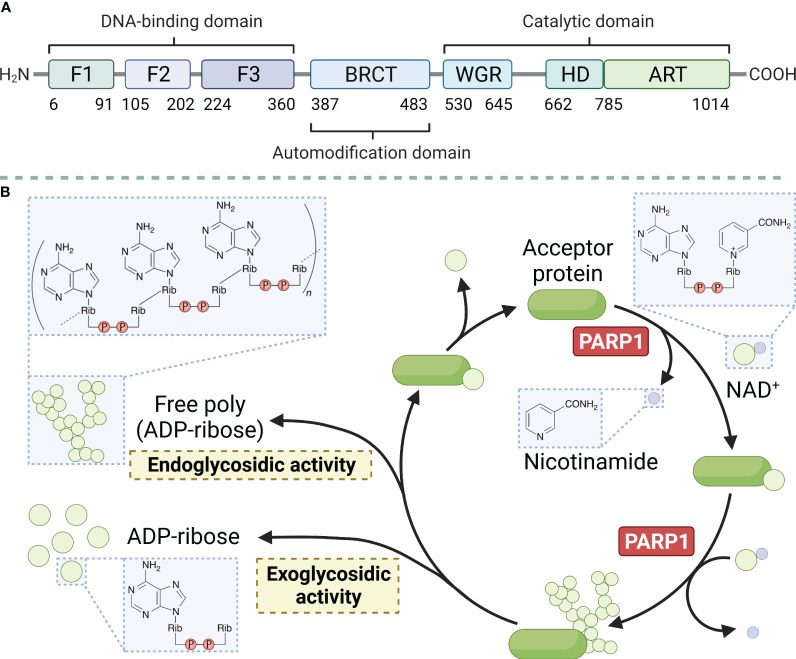
Main structural domains of PARP1 **(A)** and enzymatic PAR build-up and degradation processes **(B)**. PARP1 decomposes NAD+ into ADP-ribose and nicotinamide and attaches the ADP-ribose to a target protein, creating linear and branched PAR forms. PARG and ARH3 are the key enzymes in PAR degradation because of their exo- and endo-glycosidic actions, respectively. ADP-ribose glycohydrolases MACROD1, MACROD2, and TARG1 target mono (ADP-ribosyl)ated peptides are produced by PARG. Free PAR can also be degraded into mono (ADP-ribose).

PARP1 is a large protein with a molecular weight of approximately 113 kD. It consists of 1014 amino acids and is characterized by a modular architecture that enables its multifunctional roles in DNA repair and other cellular processes. The protein has three structural domains, namely the zinc-finger DNA-binding domain at the N-terminus, which is responsible for detecting DNA strand breaks and initiating the DNA damage response. The automodification domain in the middle, features a BRCT motif that plays a crucial role in protein-protein interactions, and the catalytic domain at the C-terminus contains the NAD^+^ binding site essential for its enzymatic activity (**Figure 1A**) (reviewed in Ray Chaudhuri & Nussenzweig, 2017) (26). PARP1 uses nicotinamide adenine dinucleotide (NAD^+^) as a donor and attaches a series of poly ADP-ribose chains to substrates, a process central to the regulation of various cellular processes including DNA repair, transcription, and cell death (**Figure 1B**) (reviewed in Curtin & Szabo, 2020) (27). Moreover, the protein possesses a regulatory domain that interacts with the catalytic domain to inhibit its activity in the absence of DNA damage. The functional diversity of PARP1 has been explored in recent years. The polymerase can undergo various post-translational modifications, such as acetylation, phosphorylation, ubiquitination, and ubiquitin-like modifications, in addition to the classical poly ADP-ribosylation (PARylation). These modifications further expand the functional repertoire of PARP1 by modulating its enzymatic activity, protein interactions, and stability (28).

#### 2.1.2 Biological functions of *PARP1*

**Figure 2:**
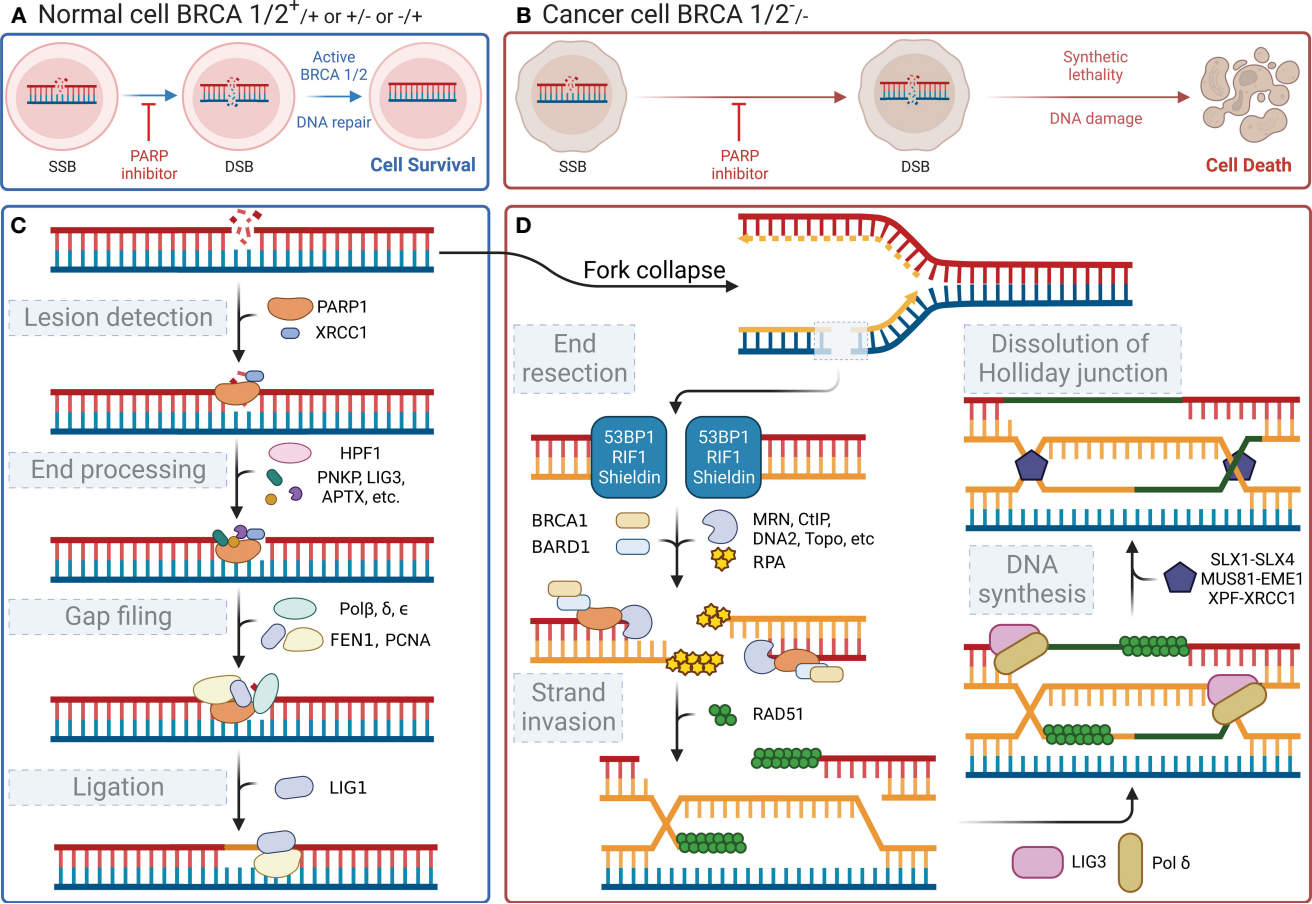
DNA repair processes and the effect of their inhibition. **(A)** Normal cells (BRCA 1/2+/+ or +/- or -/+) can activate BRCA 1/2 and initiate DNA repair after a DNA single-strand break, even when PARP inhibitors are present, ensuring cell survival. **(B)** In contrast, cancer cells (BRCA 1/2-/-) exhibit synthetic lethality and an inability to repair the DNA following a DNA single-strand break, when subjected to PARP inhibitors, ultimately leading to cell death. **(C)** When DNA single-strand damage occurs, PARP1 leads the process by identifying the damage. Then, it recruits subsequent proteins to degrade the damaged end and extend the nascent DNA. **(D)** If the function of PARP1 is disrupted, the replication fork will fail during the S/G2 phase, leading to double-strand breaks in the next replication cycle and triggering the HR repair pathway, which involves BRCA1/2. However, if BRCA1/2 is mutated, cells resort to the NHEJ pathway for repairing double-strand damage. The less reliable NHEJ pathway ultimately causes genomic instability, leading to cell apoptosis.

**Figure 3:**
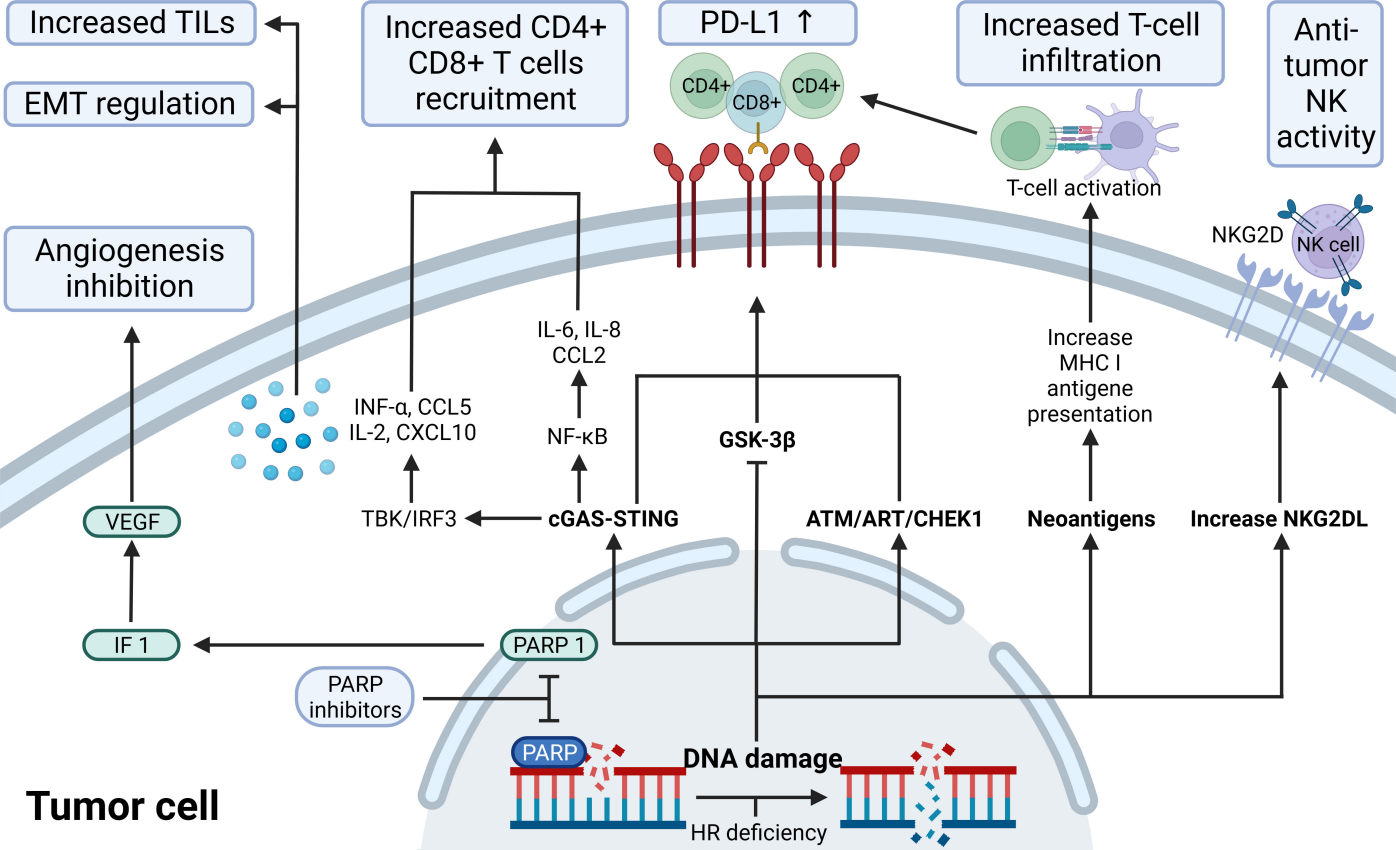
The immunological role of PARP inhibitors. The immunological role of PARP inhibitors. The major immunologic effects of PARP inhibitors include inhibiting tumor angiogenesis by modulating PARP1/HIF1-α/VEGF signaling, upregulating the release of cytokines and chemokines through the cGAS-STING/NF-k pathway, recruitingCD4+ CD8+ T, increasing TILs load and regulating EMT; upregulate PD-L1 expression through cGAS-STING, ATM-ATR-CHK1, and GSK-3β; promote antigen presentation that promotes T-cell infiltration by neoantigens associated with ICD; increase NKG2DL favoring anti-tumor NK activity. ATM, indicates ataxia telangiectasia-mutated gene; ATR, ataxia telangiectasia and Rad3 related; CCL2/5,C-C motif chemokine ligand 2/5; cGAS, cyclic GMP-AMP synthase; CHEK1, checkpoint kinase 1; CXCL10, C-X-C motif chemokine ligand 10; EMT, epithelial-mesenchymal transition; GSK-3β, glycogen synthase kinase-3β; HIF-1α,hypoxia-inducible factor-1 α; HR, homologous recombination; IL, interleukin; INF, interferon; MHC I, major histocompatibility complex class I; NF-κB, nuclear factor-kB; NKG2D(L), natural killer cells group 2D (ligand); PD-L1, programmed-death ligand 1; STING, stimulator of interferon genes; TILs, tumor-infiltrating lymphocytes; VEGF, vascular endothelial growth factor.

In the human body, each cell suffers approximately 100,000 single-stranded DNA damages per day (29). DNA damage within cells primarily includes SSBs and DSBs. These types of DNA damage trigger distinct cellular repair pathways, with *PARP1* playing a central role in orchestrating the response to each. This damaged DNA must be repaired promptly and accurately through finely regulated repair mechanisms to maintain normal cellular physiological functions. PARylation is a reversible modification of the PARP enzyme and other nuclear proteins and these PAR chains formed not only facilitate the recruitment of DNA repair enzymes but also serve as a signal for the activation of other cellular stress responses. PAR modifications turn over rapidly due to the activity of poly-(ADP-ribose) glycohydrolase (PARG) (30). It has been demonstrated that the loss of PARG frequently leads to stabilization of PARylation and causes resistance to PARP inhibitors (31). Single-stranded DNA damage can be repaired by a variety of enzymes, each with a specific function (32). Key molecules such as DNA ligase III, DNA polymerase β, and *XRCC1* are integral to the base excision repair (BER) pathway, in which PARP1 is involved. When cells experience SSBs or DSBs in their DNA, the DNA-binding domain of *PARP1* can recognize and bind to the damaged sites. Then, its catalytic domain facilitates the PARylation reaction. PARP1 acts as a substrate for itself, attaching PAR chains to its BRCT domain, which, in turn, promotes the recruitment of DNA damage repair effector proteins for binding to the PAR chains, thereby initiating the repair process (33). This recruitment includes essential factors such as *XRCC1*, which acts as a scaffold for the assembly of repair complexes. Once *PARP1* dissociates, these effector proteins continue to anchor at the damaged sites and exert their function with the assistance of histone PARylation factor 1 (*HPF1*) (34). **Figure 2C** shows the four steps of the SSB repair process mediated by *PARP1*, namely lesion detection, end processing, gap filling, and ligation. The key molecules involved at different steps, including DNA polymerase β for gap filling and DNA ligase III for ligation, have been identified and described in detail (reviewed in Hübscher, Maga, & Spadari, 2002) (35). Notably, *XRCC1* provides a platform for the repair reaction (36), polynucleotide kinase/phosphatase and apurinic/apyrimidinic endonuclease 1 are involved in the excision of irregular ends (37, 38), and flap structure-specific endonuclease 1 excises small intact DNA single strands at the ends (39). In the case of DSBs, *PARP1* facilitates the initial recognition and signaling of the break, subsequently recruiting and stabilizing key factors such as the MRN complex (*MRE11*, *RAD50*, and *NBS1*), which is crucial for the initial processing of DSBs. SSB does not lead to cell death, whereas DSB is highly likely to result in cell apoptosis. DSBs are primarily repaired through non-homologous end joining (NHEJ) or homologous recombination (HR), with *PARP1* playing a modulatory role in the choice between these pathways. During the S phase of the cell cycle, when a DNA strand containing SSB sites undergoes replication, the replication fork stalls upon reaching the damaged site and awaits the repair of the SSB sites by proteins such as *PARP1* and *XRCC1*. If the cell fails to complete the SSB repair timely, the replication fork may collapse leading to the formation of DSBs (40). The primary pathways for cell repair of DSBs include HR and NHEJ. HR is highly faithful and is the preferred pathway for cellular DSB repair. However, HR requires a double-stranded DNA strand homologous to the damaged strand as a template; therefore, it only occurs during the S/G2 phases (41). HR repair also encompasses four steps, namely end resection, strand invasion, DNA synthesis, and dissolution of the Holliday junction (**Figure 2D**) (42–44). Similar to its role in SSB repair, *PARP1* also participates in the recruitment of repair effector molecules, such as *BRCA1*, *BARD1*, and *MRE11*, in the MRN complex during DSB repair; however, *PARP1* is not essential for the functionality of these molecules (45). In addition to their synthetic lethal effect on cancer cells lacking HR, PARP inhibitors also play a crucial role in modulating both innate and adaptive immune responses (46), as illustrated in **Figure 3**.

Maintenance of genomic stability is critical for the proper functioning of organisms (47). Stalling the replication fork provides sufficient time for repairing the DNA damage. The recruitment of *PARP1* at the site of damage plays a central role in stalling the replication fork (48). Current research indicates that the recruitment of *PARP1* at the site of damage and PAR modification can suppress the activity of DNA repair-associated helicase *RECQ1*. *RECQ1* activation can restart the stalled replication forks (49), further clarifying the mechanism by which *PARP1* sustains replication fork stalling. *PARP1* can also affect the choice of DSB repair pathways and the activation of DNA damage checkpoints. The DNA damage repair process also necessitates the recruitment of various proteins. Chromatin is densely packed and wrapped around histones, which makes it less conducive for DNA damage repair; therefore, the formation of a relatively relaxed DNA structure near the damage site is required (50). Smeenk et al. (51) revealed that *PARP1* can modify nucleosome histones through PARylation, thereby promoting their dissociation. Simultaneously, the PARylation of *PARP1* can recruit various chromatin remodeling factors, such as *ALC1*, *CHD2*, *CARM1*, and *SMARCA5*. These molecules further facilitate nucleosome disassembly, which transforms the tightly wrapped chromatin into a more relaxed linear structure (52, 53). Moreover, the activation of *PARP1* can reshape chromatin structure (54) and induce programmed cell death through metabolic regulation (55).

Recent studies have elucidated the intricate interplay between PARP inhibitors and the innate immune response in tumor cells, particularly through the cGAS-STING pathway. PARP1, primarily involved in DNA repair, also suppresses PD-L1 transcription via its interaction with NPM1, as evidenced by increased PD-L1 expression following Olaparib treatment in TNBC (56, 57). This finding suggests a novel action mechanism for PARP is and supports their combination with immunotherapies. The cGAS-STING pathway, central to this interaction, is a key innate immunity component that responds to cytosolic DNA, including tumor-derived DNA, triggering inflammatory responses and antitumor immunity through the production of IFN-1 and other cytokines (58–62). PARP is, acting as STING agonists, promote T cell infiltration into tumors by accumulating double-stranded DNA in the cytoplasm and activating the cGAS-STING-TBK1-IRF3 pathway, thus inducing IFN-1 activation (63, 64). This pathway not only enhances the antitumor efficacy of radiation and immune checkpoint inhibitors but also influences the tumor microenvironment, increasing antigen presentation and cytokine production (65–68). Furthermore, PARP is induce NKG2D ligand expression in NK and tumor-specific T cells, enhancing NK cell-mediated killing and antibody-dependent cytotoxicity (69–71). The role of *PARP1* and *PARP2* in the DNA damage response underscores the potential of targeting these pathways in cancer therapy, particularly in the context of BRCA-deficient cancers (64, 72–74). This intricate relationship between PARP is and the innate immune system, especially through the cGAS-STING pathway, opens new avenues for cancer treatment, combining PARP is with other immunotherapies and highlighting the critical role of the innate immune system in cancer biology.

### 2.2 PARP1 inhibitors and their applications

#### 2.2.1 PARP1 inhibitors

**Figure 4:**
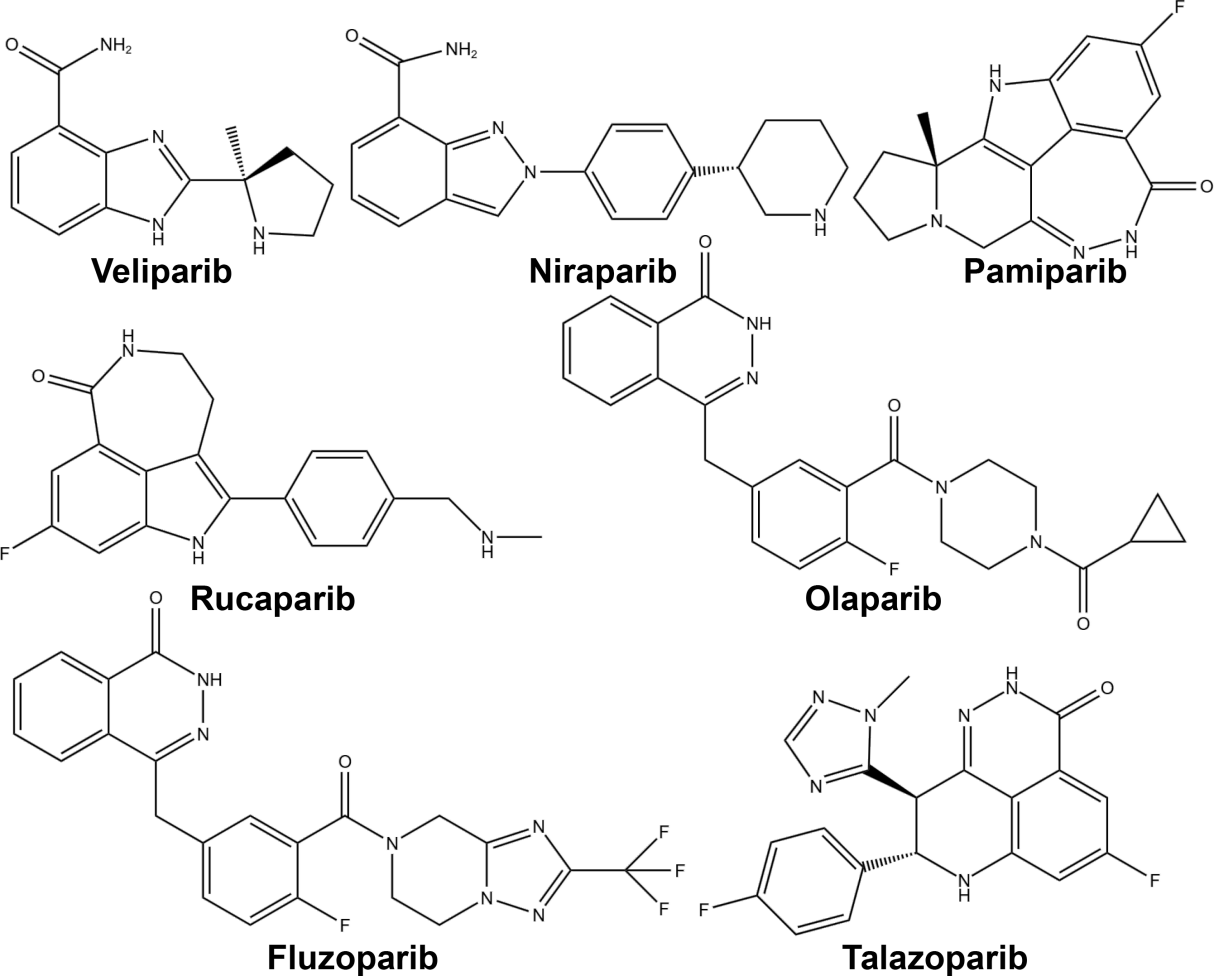
Chemical structures of major approved and clinically investigational PARP inhibitors.

PARP inhibitors have rapidly emerged as a promising approach in anticancer therapy. The second and third-generation inhibitors show improved selectivity and reduced toxicity. Several PARP inhibitors, including olaparib, rucaparib, niraparib, and talazoparib, have been approved by the United States Food and Drug Administration (FDA) and/or the European Medicines Agency. Other inhibitors, such as veliparib, are in clinical trials (**Table 1** and **Figure 4**).

**Table 1:** Major approved and investigational PARP inhibitors.

Drug	R&D Unit	Target	Indications	Status	Mean Half-Life (Hours)	Catalytic Inhibition ^1^	PARP Trapping Potency ^2^	Cytotoxicity ^3^
Olaparib	AstraZeneca	PARP1 PARP2 PARP3	Breast cancer, FTC, EOC, PPC, MPC	Approved (EU, US)	14.9 ± 8.2	6	1	259
Talazoparib	Pfizer	PARP1 PARP2	Breast cancer, NSCLC	Approved (US)	90	4	100	5
Niraparib	GSK	PARP1 PARP2	EOC, FTC, PPC	Approved (US)	36	60	2	650
Rucaparib	Clovis	PARP1 PARP2 PARP3	EOC, FTC, PPC	Approved (EU)	18 ± 1	21	1	609
Veliparib	AbbVie	PARP1 PARP2	Breast cancer, Ovarian cancer	Phase III trial	5.2	30	< 0.2	> 10,000

Catalytic Inhibition (IC50 in Wild-Type DT40 Cells; nM); ^2^, PARP Trapping Potency (Relative to Olaparib); ^3^, Cytotoxicity (EC50 in BRCA2 Mutated Capan-1 Cells; nM).

EU, European Union; EOC, Epidermoid ovarian cancer; FTC, Fallopian tube cancer; GSK, GlaxoSmithKline; PRC, People’s Republic of China; PPC, Primary peritoneal cancer; MPC, Metastatic pancreatic cancer; NSCLC, Non-small cell lung cancer; US, United States; R&D, Research and Development.

PARP1 inhibitors inhibit the process of DNA damage repair in cells by two mechanisms. First, PARP inhibitors directly suppress the enzymatic activity of PARP. When DNA damage occurs, *PARP1/2* senses DNA damage, and the zinc-finger structure in their DNA-binding domain associates with damaged DNA, thereby inducing a conformational change in them. This change alters the binding state of the cofactor NAD^+^ with ADP-ribosyl transferase, which, in turn, activates the enzymatic activity of PARP. The activated *PARP* catalyzes the cleavage of NAD^+^ into nicotinamide and ADP-ribose. Long chains of PAR form on the receptor proteins of *PARP1* (PARylation) because of the catalytic activity of ADP-ribosyl transferase. The PAR chains act as a signaling mechanism, thereby recruiting key proteins for DNA repair, such as DNA polymerase β and *XRCC1*. PARP inhibitors interact with the binding sites of NAD^+^ in the catalytic domains of *PARP1/2*, causing DNA repair failure by suppressing the enzymatic activity. Concurrently, PARP inhibitors prevent the dissociation of *PARP1* from DNA-binding sites and its autoPARylation, effectively “trapping” *PARP* at the site of damage (75, 76). This inhibition prevents DNA repair proteins from binding to the DNA nick sites and completing the repair process. Consequently, PARP-mediated SSB repair remains unfulfilled, leading to the transformation of unresolved breaks into DSBs in the subsequent replication cycle. DSBs in DNA are primarily repaired through the homologous recombination repair (HRR) pathway, which ensures high-fidelity restoration. If key factors in the HRR pathway, such as *BRCA1/2*, are mutated, cells cannot effectively initiate HRR and resort to alternative methods such as NHEJ for repair. These alternative methods do not preserve high homology in DNA and lead to erroneous repair, which results in genome instability and ultimately cell death (77). Taken together, cells lacking the capacity for HRR repair are unable to effectively repair DNA damage, and the combined effect of PARP inhibition and HRR deficiency in causing tumor cell death is known as the “synthetic lethality” effect (**Figure 2**).

Several researchers have demonstrated a significant association between *BRCA1* mutations and the development of various cancers, including pancreatic, ovarian, prostate, and breast cancers (78). Bryant et al. (8) and Farmer et al. (7) first demonstrated a “synthetic lethal” association between PARP inhibitors and mutations in *BRCA1/2* genes. The importance of the sensitivity of PARP inhibitors to *BRCA* gene status was further confirmed in subsequent clinical studies including ovarian (79), breast (80), prostate (81), and pancreatic (82) cancer patients. In recent years, the impact of germline *BRCA* status on PARP inhibitor sensitivity has also been reported in leukemia (83), renal cell carcinoma (84), and nasopharyngeal carcinoma (85). In 2020, a translational study on breast cancer suggested that olaparib was equally effective in patients with advanced breast cancer having somatic *BRCA* mutations, further suggesting that PARP inhibitors are not limited to germline *BRCA* mutations (86). In addition, Cai (87) found that the proline isomerase Pin1 can regulate *BRCA1* and make cancer cells more sensitive to PARP inhibitors, which provided a new direction for drug development targeting *BRCA*. The deprivation of *ERCC6L2*, which is an additional NHEJ component, has also been shown to restore DNA end-resection, resulting in partial restoration of HR and resistance to PARP inhibitors in *BRCA1*-deficient cells (88).

As indicated in **Table 1**, all PARP inhibitors can impede the catalytic activities of *PARP1* and *PARP2*, however, they exhibit varying degrees of cytotoxicity. The toxicity induced by PARP inhibitors significantly exceeds that caused by the deletion of *PARP* genes, implying that their anticancer properties stem from more than just inhibiting PARP’s catalytic function (89). This disparity is attributed to a phenomenon known as PARP trapping, which refers to the capacity of PARP inhibitors to stabilize PARP-DNA complexes, thereby enhancing the affinity of PARP for DNA. As evidenced in **Table 1**, the cytotoxicity of each PARP inhibitor is linked to its PARP trapping potency. Specifically, talazoparib, which has the most potent PARP trapping effect, is also the most cytotoxic. Consequently, the concept of PARP trapping should be taken into account when employing PARP inhibitors in clinical trials (90). Variations in PARP trapping efficiency may influence both monotherapy and combination therapies differently. Furthermore, the interaction of each drug with different combinatory agents varies.

#### 2.2.2 PARP1 inhibitors combined with chemotherapy

The combined application of PARP1 inhibitors and chemotherapy drugs primarily utilizes the effect of synthetic lethality. PARP1 inhibitors kill tumor cells after chemotherapy drugs have damaged the cell’s DNA by interfering with DNA damage repair. Chemotherapeutic drugs used in combination with PARP1 inhibitors mainly include alkylating agents, such as temozolomide (91, 92) and melphalan (93, 94); platinum-based drugs, such as cisplatin (95), carboplatin (96), and oxaliplatin (97); topoisomerase inhibitors, such as camptothecin (98) and irinotecan (99); anthracyclines, such as doxorubicin (100); antimetabolic chemotherapy drugs, such as gemcitabine (101); proteasome inhibitors, like bortezomib (102), and so on. Combined treatment generally benefits tumor control, and some combinations can effectively prolong the progression-free survival of cancer patients. However, in some cases, the dosage may be limited due to overlapping toxicity caused by the combination of drugs. Moreover, patients may develop drug resistance; hence there is an urgent need to develop targeted drug administration plans to improve the efficiency of medication.

#### 2.2.3 PARP1 inhibitors combined with radiotherapy

Tumor cells have a high mutation rate, are susceptible to ionizing radiation damage, have a short cell cycle block after injury, and have a short time available for DNA repair (103). By delaying and inhibiting the DNA damage repair pathways BER, HR and NHEJ, and up-regulating the expression of pro-apoptotic proteins such as Bax proteins and Bcl-2 proteins, PARP inhibitors can have a sensitizing effect on radiotherapy, especially in the phase of rapid cell growth (G2/M phase) (104–107). This effect has been confirmed in cellular experiments targeting ovarian, breast, lung, bile duct and prostate cancers (106–111). The sensitizing effect of PARP inhibitors was found to be more pronounced in the vast majority of cells carrying *BRCA* defects (111), while the location of the *p53* gene mutation (112) and the expression of the Bel-2 protein (108) were also predictive of the sensitivity of the cells to combination therapy. In studies such as the one by Kötter et al. (105), it has been observed that tumor cells shift their double-strand break (DSB) repair mechanism towards a PARP1-dependent end-joining (PARP1-EJ) pathway when treated with Olaparib. This pathway, also referred to as Alt-EJ, is specifically predominant in tumor cells. When this shift in DSB repair occurs, the tumor cells exhibit an increased sensitivity to the combined treatment of radiotherapy and Olaparib. In addition, there is a dose-dependent effect of sensitization (113). However, high doses of radiation may induce secondary tumor growth, and PARP inhibitors may also have off-target effects, leading to increased toxicity of the combination therapy on normal cells. BOURTON et al. (114) found that normal tissues such as those heterozygous for *BRCA1* may be toxicly damaged by a single insufficient dose of DNA damage repair genes. DNA damage repair-related genes such as the *ATM* gene, *p53* gene and *PRKDC* genes are mutated, individuals may also experience normal tissue toxicity after radiotherapy due to defective cell cycle checkpoint control. Therefore, further studies are needed to circumvent possible normal tissue toxicity in specific tumor types. PARP inhibitors can also be used as scaffolds for radiopharmaceuticals for internal irradiation radiotherapy, precisely targeting to tumor tissues with high *PARP* expression. On the basis of the structure of Olaparib, both ^131^I-PARPi and ^123^I-MAPi, synthesized with 131 and 123 nuclide labelling, respectively, significantly prolonged the survival time of mice in glioblastoma mouse models and showed better targeting properties (115, 116). In addition, olaparib (117) and lucaparib (118), labelled with ^125^I nuclides emitting Rusche electrons, respectively, showed good targeting and killing effects on triple-negative breast and ovarian cancer cells, respectively, in *in vitro* cellular assays. Internal irradiation radiotherapy can be precisely targeted to tumor tissues to exert drug effects, but fewer studies in this area have been reported so far, which deserves more in-depth exploration. To date, dozens of clinical trials combining PARP inhibitors with radiotherapy are registered, which mainly involve testing of veliparib or olaparib (**Table 2**) (119).

**Table 2:** Published clinical studies on PARP inhibitors combined with radiotherapy.

Treatments	Cancer	Phase	Efficacy	Identifier
Veliparib + RT	Breast cancer	I	3-year OS 56.6%, PFS 50%	NCT01477489
Veliparib + Capecitabine + RT	Rectal cancer	I	Tumor downstaging after surgery-71%; pCR 29%	NCT01589419
Veliparib + RT	Gliomas	I/II	Phase I: PR 0% SD 91.7% PD 8.3% Phase II: PR 13.2% SD 71.7% PD 9.4%	NCT01514201
RT + Temozolomide +/− Veliparib	Glioblastoma	II	Without veliparib PFS at 6 months 31% Median OS:12.8 months With veliparib PFS at 6 months 46% Median OS:12.7 months	NCT02152982
Olaparib + RT + Cetuximab	HNSCCs	I	Median OS: 37 months 2-year OS (72%), PFS (63%)	NCT02308072

HNSCCs, head and neck squamous cell carcinoma; OS, overall survival; pCR, pathological complete response; PD, progressive disease; PFS, progression free survival; PR, partial response; RT, radiotherapy; SD, stable disease.

#### 2.2.4 PARP1 inhibitors combined with immunotherapy

The occurrence and development of tumors are closely related to the dysfunction of human immune function. Immune checkpoints can suppress T cell function under normal circumstances, but can be exploited in tumor tissues to form immune escape (120). Currently, immune checkpoints identified by researchers include programmed death protein-1 (PD-1), cytotoxic T-lymphocyte-associated antigen 4, and programmed death ligand-1 (PD-L1) (120). Immune checkpoint inhibitors can inhibit the activity of the above molecules, activate T cells’ immune response to tumors, and thus exert anti-tumor effects. The theoretical basis for the combined use of PARP inhibitors and immunotherapy is mainly based on two assumptions: first, PARP inhibitors can increase tumor mutation burden and thereby increase the production of new antigens, stimulating anti-tumor immune response when treating HRD tumors; second, PARP inhibitors can activate innate immune responses by activating the STING pathway and upregulating PD-L1 expression, thus enhancing anti-tumor effects (121). In human colorectal cancer, human lung squamous cell carcinoma, human breast cancer, human sarcoma, and human bladder cancer cells, Wang et al. (122) found that the combination of niraparib and PD-1 inhibitors can increase immune cell infiltration and delay the growth of tumor cells, and this combined effect is independent of BRCA gene typing. Ding et al. (121) found that in BRCA1-deficient ovarian cancer mice, olaparib can trigger local and systemic anti-tumor immune responses, activate the STING pathway and upregulate PD-L1 expression; and when combined with PD-1 inhibitors, this immune triggering effect is further enhanced, resulting in stronger inhibition of tumor cell growth in nude mice, significantly prolonging the survival of the mice. In addition, ongoing phase 3 studies combining PARP inhibitors with immunotherapy are summarized in **Table 3**.

**Table 3:** Major phase III clinical trials combining PARP inhibitors with immunotherapy.

Drug	Cancer	Immunotherapy	Trial ID	Identifier
Olaparib	Ovarian cancer	Pembrolizusmab	ENGOT-OV43/KEYLYNK-001	NCT03740165
	Ovarian cancer	Durvalumab	DUO-O	NCT03737643
	Endometrial cancer	Durvalumab	DUO-E	NCT04269200
	TNBC	Pembrolizumab	KEYLYNK-009	NCT04191135
	NSCLC	Pembrolizumab	KEYLYNK-006	NCT03976323
	NSCLC	Pembrolizumab	KEYLYNK-012	NCT04380636
	NSCLC	Pembrolizumab	KEYLYNK-008	NCT03976362
	mCRPC	Pembrolizumab	KEYLYNK-010	NCT03834519
Talazoparib	Ovarian cancer	Avelumab	JAVELIN ovarian PARP 100	NCT03642132
Niraparib	Ovarian cancer	Dostarlimab	ENGOT-0V44/FIRST	NCT03602859
	Ovarian, fallopian tube or primary peritoneal cancer	Dostarlimab	NItCHE-MITO33	NCT04679064
	Ovarian, tubal or peritoneal cancer	Atezolizumab	ANITA	NCT03598270
	Ovarian carcinosarcoma	Dostarlimab	ROCSAN	NCT03651206
	Endometrial cancer	Dostarlimab	RUBY part 2	NCT03981796
	NSCLC	Pembrolizumab	ZEAL-1L	NCT04475939
Rucaparib	Ovarian cancer	Nivolumab	ATHENA	NCT03522246

mCRPC, metastatic castration-resistant prostate cancer; NSCLC, non-small cell lung cancer; TNBC, triple negative breast cancer.

Moreover, initial studies in experimental models have shown that combining radiotherapy, PARP inhibitors, and immunotherapy leads to better tumor infiltration and enhances antitumor immune responses mediated by T cells in mouse models. The combined use of sub-ablative radiotherapy, olaparib, and anti-PD-1 was more effective in inhibiting tumor growth than single or dual therapies in both microsatellite stable and unstable colon cancer (123). In a small-cell lung carcinoma (SCLC) mouse model, the use of radiotherapy, niraparib, and anti-PD-1 led to an increase in median survival time and a decrease in tumor volume (124). Numerous clinical trials in phases I to III are investigating various combinations of radiotherapy, PARP inhibitors, and immunotherapy in cancer treatment (125). These trials often include at least one group receiving these three treatments either together or in sequence, sometimes along with standard chemotherapy. The effectiveness of combining PARP inhibitors with radiotherapy and immunotherapy, which targets CTLA-4 and/or PD-1/PD-L1 pathways, will be evaluated in various cancers including NSCLC, SCLC, breast, prostate, pancreatic, gastroesophageal, rectal, and head and neck carcinomas (125). Many of these trials are currently in the recruitment phase or not yet active, with results expected in the coming years (**Table 4**) (125).

**Table 4:** Recently approved clinical trials using combinations of radiotherapy, PARP inhibitors, and immunotherapy.

Treatments	Cancer	Phase	Estimated completion dates	Identifier
Durvalumab, Olaparib, RT	Pancreatic cancer	I	Primary and final: 31 March 2024	NCT05411094
Niraparib, Dostarlimab, RT	Rectal cancers	I/II	Primary: 31 December 2024 Final: 31 December 2026	NCT04926324
Niraparib, Dostarlimab, RT	Pancreatic cancer	II	Primary: 19 January 2022 Final: October 2026	NCT04409002
Niraparib, Dostarlimab, RT	Breast cancer	II	Primary: 1 April 2023 Final: 1 December 2029	NCT04837209
Durvalumab, Tremelimumab, Olaparib, RT	SCLC	I	Primary and final: 1 June 2023	NCT03923270
Pembrolizumab, Olaparib, RT	Breast cancer	II	Primary and final: January 2025	NCT04683679
Pembrolizumab, Olaparib, ADT, RT	Prostate cancer	II	Primary: 2 January 2025 Final: 2 January 2028	NCT05568550
Carboplatin, Durvalumab, Etoposide, Olaparib, RT	SCLC	I/II	Primary and final: 31 January 2024	NCT04728230
Pembrolizumab, Olaparib, RT	Gastric cancers	II	Primary: December 2025 Final: December 2028	NCT05379972
Pembrolizumab, Olaparib, Etoposide, Platinum, RT, PCI	SCLC	III	Primary and final: 28 October 2027	NCT04624204
Pembrolizumab, Olaparib, Cisplatin, RT	HNSCCs	II	Primary: 31 October 2024 Final: 31 October 2025	NCT05366166
Pembrolizumab, Olaparib, Etoposide, Carboplatin, Cisplatin, Paclitaxel, Pemetrexed, RT, Durvalumab	NSCLC	III	Primary and final: 6 July 2026	NCT04380636

ADT, androgen deprivation therapy; HNSCCs, head and neck squamous cell carcinoma; SCLC, small-cell lung cancer; PCI, prophylactic cranial irradiation; NSCLC, non-small-cell lung cancer.

## 3 Effects of PARP and its inhibitors on antitumor immunity

### 3.1 The impact of PARP and its inhibitors on T cells

T cells are an integral part of the immune system, essential in combating infections and cancer cells. Both *PARP1* and *PARP2* proteins are expressed in thymic cells, but only *PARP2* plays a significant role in the development of thymic cells. The knockdown of *PARP2* in mice results in a reduction of CD4^+^ CD8^+^ thymic cells, likely related to *PARP2*’s role in inhibiting DNA double-strand break accumulation-induced apoptosis (126). Specific knockdown of *PARP2* in T cells of *PARP1*-deficient mice disrupts T cell homeostasis, reducing CD4^+^ and CD8^+^ T cells, especially CD8+ T cells, with an increase in DNA damage and apoptotic markers. This suggests that the reduction in T cells may be due to the accumulation of genetic mismatches leading to cell death, rather than solely maturation barriers (127). Compared to mice with single deficiencies in T cell *PARP1* or *PARP2*, mice with *PARP1/2* double deficiencies have faster proliferating breast cancer cells and reduced CD4^+^ and CD8^+^ T cell infiltration in tumor tissues (128).

*PARP* also plays a crucial role in T cell differentiation. *PARP1* can affect the differentiation of CD4^+^ T cells by activating the nuclear factor NFAT (129). The lack of *PARP1* can lead to a reduced expression of NFAT-dependent cytokines (such as IL-2 and IL-4), further affecting the differentiation of immune cells (130). The absence or inhibition of *PARP1* may bias CD4+ T cells towards type 1 T helper cell (Th1) differentiation (131). *PARP1* can regulate the expression of the transforming growth factor β (TGF-β) receptor in CD4^+^ T cells, and in the absence of PARP1, continuous stimulation with TGF-β1 and IL-6 enhances the ability of CD4^+^ T cells to differentiate into Th17 cells *in vitro* (129). In an asthma mouse model, Olaparib increases the expression of Th1-related cytokines such as interferon (IFNγ) and transcription factor T-bet, while inhibiting the expression of Th2-related cytokines such as IL-4, IL-5, IL-6, IL-13, and macrophage colony-stimulating factor (M-CSF) (128). In an arthritis mouse model, PARP inhibition is associated with a reduction in Th1-related cytokines tumor necrosis factor α (TNFα) and IFNγ expression and can inhibit the proliferation of some Th1 cells (132).

In addition to affecting T cell differentiation, *PARP1* and *PARP2* also impact T cell function. In *BRCA*-deficient ovarian cancer, triple-negative breast cancer, and small cell lung cancer mouse models, Olaparib may activate the cGAS-STING pathway, upregulate *CXCL10* and *CCL5* expression, recruit CD8^+^ T cells to tumor tissue, and activate antitumor immune responses (133). Olaparib can also reduce the expression of T cell immune checkpoint receptors PD-1, Tim-3, and Lag-3, which are associated with T cell inhibition and exhaustion (121). The forkhead box protein 3 (*Foxp3*) is one of the key transcription factors controlling the development and immunosuppressive function of regulatory T cells (Tregs) (134). Luo et al.’s research indicates that *PARP1* can interact with Foxp3 and induce its poly ADP-ribosylation (135). In model mice with a single deficiency in T cell *PARP1* or *PARP2*, there is no change in the number of T cells in peripheral lymphoid tissues (136), but the double deficiency of *PARP1* and *PARP2* leads to a reduction in peripheral CD4^+^ CD8^+^ T cells in breast cancer model mice (129). These results suggest that PARP inhibitors are related to the activation of effector T cells.

### 3.2 The impact on tumor-associated macrophages (TAM)

TAMs are important immune cells constituting the tumor microenvironment. Macrophages are highly heterogeneous and can adapt to the tumor microenvironment by changing their immune phenotype, including the protumor phenotype M2 type and the antitumor phenotype M1 type (137). The main function of M1 type is to drive Th1 response and has cytotoxicity against microbes and tumor cells. The M2 type (also known as alternatively activated macrophages) can be activated by IL-4 or M-CSF, produce arginase-1, decompose arginine into ornithine, promote the formation of the extracellular matrix, and facilitate tumor invasion and metastasis. M2 type macrophages can also inhibit inflammation by secreting anti-inflammatory factors such as IL-10, participate in tumor angiogenesis and extracellular matrix remodeling by secreting angiogenesis-related factors, control inflammatory responses by downregulating M1 type macrophage-mediated functions and adaptive immunity, and play a protumor role (138). Macrophages have the potential to phagocytose tumor cells and present tumor-specific antigens to induce adaptive antitumor immunity (139). Targeting TAMs has become a potential new strategy for tumor immunotherapy.

The metabolic pathways of macrophages are closely related to their phenotype and function, with M1 type mainly showing enhanced glycolysis and elevated glutathione levels, and M2 type showing enhanced fatty acid oxidation (140). The characteristics of macrophages make them easily affected by various stresses and damages. PARP1 can protect macrophages from oxidative-induced death and can inhibit the transcription of IL-6 by interacting with the MLL1 methyltransferase (141, 142). In *BRCA*-deficient breast cancer, PARP inhibitors can affect the metabolism of TAMs, thereby affecting their degree of immunosuppression (143). TAMs may inhibit PARP inhibitor-induced tumor cell DNA damage, thereby weakening sting-dependent antitumor immunity (144). However, there are currently few reports on the direct effects of PARP inhibitors on macrophages, and the mechanisms of action are not clear.

### 3.3 The impact on dendritic cells

DCs are key antigen-presenting cells in the tumor microenvironment and can activate and induce T cell differentiation (145). Olaparib can significantly increase the proportion of mature DCs and enhance their antigen-presenting ability (146). In *BRCA*-deficient ovarian cancer and triple-negative breast cancer mouse models, Olaparib can activate the cGAS/STING pathway in tumor cells, thereby activating TBK1/IRF3 signaling in DCs (146). In addition, compared to mice with a single deficiency in *PARP1*, a single deficiency in *PARP2*, or normal control mice, breast cancer mice with *PARP1/2* double deficiencies have a higher percentage of CD11b^+^DCs in cancer tissues (129). However, the above conclusions are all indirect effects of PARP and its inhibitors on the recruitment and function of DCs, and there is currently no direct evidence of their impact on DC function. Interestingly, in a mouse model of autoimmune encephalomyelitis, PARP inhibitors can directly inhibit the activation of NF-κB and maturation of mouse bone marrow-derived DCs, thereby affecting the migration and antigen-presenting function of DCs, reducing the severity and recurrence rate of the disease (147).

### 3.4 The impact of PARP and its inhibitors on NK cells

NK cells play a key role in antitumor immune responses by killing tumor cells before antigen exposure. Tumor necrosis factor-related apoptosis-inducing ligand (TRAIL) is a key effector molecule of NK cells, proven to prevent tumor occurrence, growth, and metastasis (148). Olaparib and Veliparib can upregulate the expression of pro-apoptotic molecules Fas and death receptor 5 (DR5) on the surface of various cancer cells (leukemia, ovarian cancer, and lung cancer cells, etc.), making cancer cells more sensitive to TRAIL-induced apoptosis (149). Olaparib may enhance the sensitivity of prostate cancer cells, whether *BRCA* wild-type or mutant, to NK cells by upregulating the expression of the death receptor TRAIL-R2 on the surface of prostate cancer cells (70). In a mouse model of human acute myeloid leukemia transplantation, the knockout or inhibition of *PARP1* can induce the expression of NKG2D ligands on drug-resistant leukemia stem cells. These ligands are key mediators of NK cell antitumor immunity, thereby promoting NK cells to target and eliminate leukemia stem cells and inhibit leukemia occurrence (150). This evidence suggests that PARP inhibitors have a place in NK cell-mediated antitumor immunity.

### 3.5 The impact of PARP and its inhibitors on B cells

B cells play a crucial role in tumor immunity, with their protumor or antitumor effects depending on the tumor microenvironment (151). *PARP* can affect B cell homeostasis. Although the double deficiency of *PARP1/2* does not affect the number of mouse bone marrow B progenitor cells and peripheral mature B cells, it leads to a reduction in transitional and follicular B cells in peripheral blood, suggesting that *PARP* may play an important role in B cell differentiation (152). The V(D)J gene rearrangement of immunoglobulins is essential for the production of immunoglobulins in pre-B cells. DNA double-strand breaks caused by V(D)J rearrangement can be repaired by the *PARP1*-mediated NHEJ pathway (153). The double deficiency of B cell *PARP1/2* does not affect V(D)J rearrangement but leads to reduced serum IgG response levels to non-T cell antigens (148). *PARP* can also inhibit the differentiation and maturation of B cells in germinal centers (154). However, it is still unclear how PARP inhibitors affect B cell homeostasis and immunoglobulin production in solid malignancies.

## 4 Advances in the use of PARP inhibitors for radiotherapy sensitization

The development of PARP1 inhibitors has a rich history. The first PARP1 inhibitor, 3-aminobenzamide, was developed as early as 1979 with the aim of enhancing sensitivity to alkylation agents (155) and to increase radiation-induced cytotoxicity (10). This use of PARP inhibitors as chemosensitizing and radiosensitizing agents persisted until the concept of synthetic lethality was published in 2005 (7, 8). Following this, their use was extended to monotherapy. However, monotherapy has been shown to possess many adverse effects and can present significant limitations in certain cases (156, 157). An understanding of the role of *PARP1* in DNA damage repair has prompted several researchers to combine it with conventional chemotherapeutic agents and radiotherapy to enhance the overall efficacy while reducing the toxicity of each treatment type. The sensitizing effect of PARP inhibitors on chemotherapy has been initially validated in clinical practice, and better clinical efficacy has been obtained (reviewed in Wang, Chen & Do, 2021) (158). To date, the results of several preclinical and clinical studies have suggested that *PARP1* can act as a radiosensitization target in different types of tumors.

### 4.1 Preclinical studies

In a seminal study, Chalmers et al. (159) reported that PARP inhibitors enhanced the sensitivity of glioma cells to radiotherapy. The authors also revealed that the sensitizing effect of PARP inhibitors on radiotherapy was associated with the specific phase of the tumor cell cycle at the time of irradiation. Calabrese et al. (160) observed that the combination of radiation and PARP inhibitors significantly reduced the survival rate of colorectal cancer cells. In their study, they established a xenograft model by subcutaneously implanting tumor cells into nude mice. Interestingly, after a 30-minute irradiation with 2 Gy X-rays followed by treatment with the PARP inhibitor (AG14361), they noticed a delay in the growth of LoVo xenograft tumors. Furthermore, they observed a regression of SW620 xenograft tumors. It’s essential to emphasize that the PARP inhibitor sensitized the tumors to the radiation, and not the other way around. Additionally, this combination treatment did not lead to any severe side effects.

Subsequently, the radiosensitizing effect of PARP inhibitors gradually gained attention. Zhan et al. (18) conducted a study on the influence of the PARP inhibitor AZD2281 on esophageal cancer cells under both normoxic and hypoxic conditions. The findings showed that AZD2281 significantly amplified the apoptosis triggered by radiotherapy under hypoxic conditions. This enhancement is possibly due to the chronic hypoxic ESCC cells being HR deficient, which may lead to a state of contextual synthetic lethality with the PARP inhibitor, thereby aiding in radiation sensitization. Tuli et al. (161) also indicated that the combination of PARP inhibitor ABT-888 and radiotherapy prolonged the tumor growth phase in a mouse prostate *in situ* transplantation tumor model, whereas ABT-888 or radiotherapy alone was less effective.

Feng et al. (21) showed that PARP inhibitors increased the radiosensitivity of breast cancer cells independent of *BRCA1* mutational status. Bi et al. (111) also found that the PARP inhibitor Olaparib had radiosensitizing effects on both *BRCA1*-normal and -mutated high-grade plasmacytotic ovarian cancer cell lines, and the effect was more pronounced in *BRCA1*-mutated cells. Additionally, it was reported that tumors mutated due to the *BRCA1* gene developed resistance as a result of *BRCA1*-independent homologous recombination restoration which can be sensitized to radiotherapy (162). These findings suggest that PARP inhibitors have potential applications as radiosensitizers in several tumor types, including cholangiocarcinoma, melanoma, head and neck squamous cell carcinoma, and soft tissue sarcoma (163–166).

The mechanism by which PARP inhibitors exert radiosensitizing effects is intricately connected to the repair of single-strand DNA breaks (SSBs) induced by ionizing radiation. Further research continues to explore the intricate details and additional potential mechanisms underlying this radiosensitization process. Laird et al. (167) from Memorial Sloan Kettering Cancer Center, USA used two different PARP inhibitors at the same concentration of enzyme inhibition and found that veliparib had no radiosensitizing effect, whereas talazoparib showed marked radiosensitizing effects. Subsequent studies have shown that inhibitors with a higher *PARP* trapping capacity induce more DNA DSBs, thereby enhancing the radiosensitization of tumor cells.

### 4.2 Clinical studies

Several researchers are conducting clinical studies of PARP inhibitors in combination with radiotherapy based on the favorable results of preclinical studies, and preliminary findings are positive. In 2018, Karam et al. published the results of phase I clinical trial, which was conducted to investigate the safety and toxicity of PARP inhibitor olaparib combined with cetuximab in patients with head and neck squamous cancer treated with radiotherapy (NCT01758731) (168). Sixteen patients received oral olaparib daily (25–200 mg twice/day), cetuximab on the third day of initiation (initial dose 400 mg/m²), and then radiation therapy (69.3 Gy/33 doses) after 5–7 days. Patients had two-year overall survival, progression-free survival, and local control rates of 72%, 63%, and 72%, respectively. The most common treatment-related grade 3-4 adverse effects were radiation dermatitis and mucositis, reported in 38% and 69% of patients, respectively. The maximum tolerated dose was 50 mg twice daily. Overall, combination therapy was potentially beneficial in terms of overall survival of patients, and the authors confirmed the efficacy and safety of PARP inhibitors in combination with radiotherapy.

In 2019, Cedars-Sinai Medical Center published the results of another clinical trial in patients with locally advanced pancreatic cancer (NCT01908478) (169), which was conducted to investigate the effects of veliparib in combination with gemcitabine and radiotherapy in 30 patients. Patients received weekly gemcitabine (1000 mg/m², intravenous) and concurrent intensity-modulated radiation therapy (36 Gy/15 doses) and veliparib (initial dose 20 mg twice/day) for 3 weeks. The median progression-free and overall survivals of 30 patients were 9.8 months (95% CI: 8.4–18.6) and 14.6 months (95% CI: 11.6–21.8), respectively. In addition, high *PARP3* and low *RBX1* levels were associated with improved overall survival of patients. Grade 3 or higher adverse reactions during treatment mainly included lymphopenia and anemia, and the most common adverse reactions were gastrointestinal reactions, hematologic toxicity, and fatigue.

A phase I study was conducted at the University of Maryland School of Medicine (NCT00649207) for evaluating veliparib in combination with whole-brain radiotherapy (WBRT) in patients with brain metastases (170). Eighty-one patients (median age: 58 years) participated in the study, and the primary tumor types were predominantly non-small cell lung cancer (34 patients) and breast cancer (25 patients). All patients received WBRT (30.0 Gy/10 doses or 37.5 Gy/15 doses) in combination with veliparib (doses ranging from 10–300 mg twice/day) to determine the maximum tolerated dose and the recommended corresponding therapeutic dose. Grade 3 or 4 adverse reactions that may be associated with veliparib were fatigue (30%), nausea (22%), decreased appetite (15%), and vomiting (14%). Preliminary efficacy results suggested that the median survival time of patients was 10.0 months (95% CI: 3.9–13.5) and 7.7 months (95% CI: 2.8–15.0) in the non-small cell lung cancer and breast cancer groups, respectively. The team is conducting a randomized controlled phase IIb study based on these promising results.

In sum, the preliminary results of the studies on the combination of PARP inhibitors with radiotherapy have provided an interesting direction for future research. These initial findings suggest potential benefits in terms of progression-free survival and overall survival. However, it is important to note that these studies have also shown that there is sometimes an increase in toxicity, including late toxicity. Therefore, it is essential to consider a balanced perspective on the available evidence, particularly as the field anticipates further data from advanced clinical studies on this combination therapy in the next 3–5 years. It is hoped that these forthcoming studies will provide a more definitive understanding of the safety and efficacy of combining PARP inhibitors with radiotherapy.

## 5 Mechanism of action of PARP1 inhibitors as radiosensitizers

PARP inhibitors have demonstrated excellent antitumor effects in several large phase III clinical trials. Currently, these inhibitors are clinically approved by the FDA for treating several malignancies, including ovarian, breast, and prostate cancers with *BRCA* mutations. In addition, preclinical data indicated that PARP inhibitors can increase the radiosensitivity of tumor cells, and clinical trials of PARP inhibitors in combination with radiotherapy are being conducted (24). However, the specific mechanism by which PARP1 inhibitors exert radiosensitization effects is still unclear. We summarized the main concepts in subsequent sections.

### 5.1 Replication period sensitization

The sensitivity of cells to radiation is different at different stages of the cell cycle. Cells in or near the M phase have the highest sensitivity to radiation followed by G2 and M phase cells; however, late S-phase cells show greater resistance to radiation compared with others. Jain et al. (171) conducted a comparative study on the radiosensitizing effect of PARP inhibitors on human and rodent tumor cells. They found that the radiosensitizing effect of PARP inhibitors was evident only in S-phase cells. The radiosensitizing effect of PARP inhibitors on human tumor cells was less pronounced than that on rodent tumor cells because of the longer accumulation of G2/M and G1 phases in human cells and the relatively shorter exposure of S-phase cells to radiation. Dungey et al. (172) explored the radiosensitivity of glioblastoma cells and found that PARP inhibitors had the most significant radiosensitizing effect on S-phase glioblastoma cells (SER50 = 1.60) compared with G1-phase (SER50 = 1.27) and G2-phase (SER50 = 1.33) glioblastoma cells. Follow-up studies confirmed the radiosensitizing effect of PARP1 inhibitors on S-phase cells not only in fibroblasts carrying capillary ataxia mutation genes (173) but also in human lung, breast, glioma, and head and neck cancer cells (106, 172, 174, 175). The main mechanism involves induction of apoptosis by inhibiting the repair of damaged DNA in replication-phase cells and exacerbating DNA damage. Notably, endogenous *PARP1* silencing was superior to the application of PARP1 inhibitors for enhancing radiosensitivity (176).

Löser et al. (173) found a replication-independent radiosensitization in DNA ligase IV-deficient fibroblasts. Similarly, Kötter et al. (105) compared different cell lines and reported that *PARP1* inhibitors enhanced cellular radiosensitivity in a replication-independent manner. Notably, inhibition of DNA replication using the DNA polymerase inhibitor aphidicolin did not affect the radiosensitizing effect. The researchers further determined that DNA repair in these cells shifted from the classical NHEJ pathway to a less precise selective end-joining pathway dependent on *PARP1*; the results were in agreement with those of their previous study (177). More recently, Oing et al. (108) found that prostate cancer cells overexpressing the B lymphocytoma 2 (*Bcl-2*) gene also rely on this selective end-joining pathway for repair and show radiosensitivity to PARP1 inhibitors. In addition, overexpression of the *Bcl-2* gene may be a factor driving the shift from an NHEJ pathway to a selective end-joining pathway (108). This finding opens up new possibilities for the use of PARP1 inhibitors in cancer therapy (108).

### 5.2 Other potential radiotherapy sensitization mechanisms

The cellular response to ionizing radiation is highly dependent on the presence of oxygen. Most solid tumors tend to form hypoxic zones, and local hypoxia, a prominent feature of the tumor microenvironment, is thought to be one of the main causes of tumor resistance to radiotherapy. The combination of PARP inhibitors with radiotherapy can alleviate the resistance to radiation triggered by hypoxia. For example, PAR*P* inhibitors, such as olaparib, cause vasodilation and increase tumor perfusion, effectively ameliorating hypoxia and areas of resistance to radiation within the tumor, thereby enhancing tumor sensitivity to radiation (178). Moreover, PARP inhibitors still exhibit radiosensitizing effects in cells in hypoxic environments in the absence of vascular system improvement (179), possibly because of genetic instability resulting from mutational phenotypic effects induced by hypoxia. This effect is associated with a reduction in the transcription of HRR-related proteins, which leads to a synergistic effect of “synthetic lethality” (180).

Vance et al. (181) investigated the effect of a cell cycle checkpoint kinase 1 inhibitor in combination with a PARP1 inhibitor on radiosensitization for the first time. This combination application markedly enhanced the radiosensitivity of pancreatic cancer cells triggered by *p53* gene mutations while having minimal toxic and radiosensitizing effects on normal cells. The possible mechanism is through the elimination of the G2 checkpoint and inhibition of HRR, which leads to the accumulation of DNA damage. Next, the team selected a serine/threonine protein kinase 1 (*Wee1*) inhibitor with similar effects in combination with a PARP1 inhibitor and also showed enhanced radiosensitivity of pancreatic cancer cells. These results suggested that PARP1 inhibitors and drugs targeting the G2 checkpoint may synergize with radiation to produce a “synthetic lethal “ effect (182). This finding not only confirmed the radiosensitizing effect of PARP1 inhibitors but also stimulated an interest in multitargeted combined sensitization strategies. Azad et al. (183) validated the radiosensitizing effect of DNA-dependent protein kinase inhibitors by combining them with PARP1 inhibitors in non-small cell lung cancer cells and found that the main mechanism was accelerated cellular senescence.

Further, Zhou et al. (184) and Chen et al. (185) showed that PARP1 inhibitors could modulate radiation-induced protective autophagy by regulating the adenosine monophosphate-dependent protein kinase/mammalian target of rapamycin pathway. These findings confirmed that radiation-induced autophagy is a protective mechanism for tumor cells; the inhibition of PARP1 prevents autophagy, thereby increasing the sensitivity of nasopharyngeal carcinoma cells to radiation. These findings provide valuable insights into the versatility and specificity of PARP1 as a radiosensitization target in different tumor types.

## 6 Limitations and challenges

*PARP1* is a promising target for radiosensitization; however, the use of PARP inhibitors in combination with radiation therapy is still in its infancy. Despite promising preliminary clinical trial data, PARP1 inhibitors remain understudied in combination with radiation therapy, and combining PARP1 inhibitors with radiation therapy remains challenging for clinical application.

First, a deeper understanding of the mechanism of action of PARP1 is essential. PARP1 is currently known to play an important role in DNA repair; however, it is difficult to precisely define and classify its specific mechanism of action. The information on the signaling pathways involved is scarce, and a solid theoretical basis to support clinical applications is lacking. Different tumor types exhibit different radiosensitivities when combined with inhibitors and radiotherapy. Moreover, the mechanism of action of this therapeutic approach varies among tumors due to tumor heterogeneity, which also needs to be explored in depth by researchers.

Second, researchers have not confirmed the safety and efficacy of long-term use of PARP1 inhibitors. Alotaibi et al. (186) showed that the combination therapy only paused tumor tissue growth and did not trigger apoptosis of tumor cells; therefore, further studies are needed to investigate whether this combination therapy is effective in improving long-term survival. Moreover, the effect of PARP inhibitors on highly proliferative non-tumor tissues (e.g., mucosa and bone marrow) is unclear. Some authors have found that PARP inhibitors may increase the risk of myelodysplasia or acute myeloid leukemia (187). Therefore, further studies are needed to determine the optimal dose, the time window of administration, and the management of adverse effects of combination therapies. In addition, in considering the optimal radiotherapy regimen for combination with PARP1 inhibitors, both hyperfractionation and hypofractionation present potential advantages. Hyperfractionation, involving lower doses over several administrations, may allow for increased DNA damage at a rate that outpaces the cell’s ability to repair, thereby enhancing the effect of PARP1 inhibitors. On the other hand, hypofractionation, involving higher doses in fewer fractions, may induce a more substantial initial level of DNA damage, potentially overwhelming the cell’s repair mechanisms and leading to cell death. However, this approach may also increase the risk of off-target effects and toxicity. As of now, it remains unclear which regimen would be more beneficial in combination with PARP1 inhibitors. Further research, including preclinical studies and clinical trials, is needed to elucidate the optimal RT regimen for this combination therapy.

Finally, reliable biomarkers are crucial to identify patients who can benefit from such combination therapies (personalized treatment) and for predicting the efficacy of treatment. PARP1 inhibitors are effective as monotherapy for *BRCA*-deficient cancers, whereas they are effective in combination with radiotherapy or other therapeutic modalities for non-gene-deficient cancers. However, all genes associated with DNA repair or that can trigger synthetic lethality are not known, and identifying mutations in DNA damage response proteins and repair pathways may become one of the main criteria for selecting PARP1 inhibitors for cancer treatment. Alternatively, genetic characterization can be used to determine whether a particular tumor is responsive to PARP1 inhibitors. For example, Liu et al. (188) found that the radiosensitizing effect of PARP1 inhibitors was dependent on the loss of *p53* function in a bladder cancer model. This implies that *p53* may be a biomarker that can be used to predict the radiosensitizing effect of PARP1 inhibitors. Mao et al. (113) revealed that low doses of PARP1 inhibitors increased the radiosensitivity of cholangiocarcinoma cells and suggested that the radiation-induced increase in PAR can also predict radiosensitization by PARP1 inhibitors. In addition to *BRCA1/2*, the status of other genes (such as *ATM*, *ATR*, *PALB*, and *FANC*) of the HRR pathway can also be assessed to predict the efficacy of PARP inhibitors.

## 7 Conclusion and future directions

Radiation therapy has rapidly evolved over the past decades and has taken a central role in the treatment of many malignancies. However, the effectiveness of radiation therapy is limited and has not achieved the desired results in treating several types of cancer. Therefore, enhancing the efficacy of radiotherapy has become a key area of current research on cancer therapeutics (189). In this context, radiotherapy-sensitizing drugs (represented by glycididazole sodium) have emerged in clinical oncology treatment. These drugs can enhance the sensitivity of tumor cells to radiation and are minimally toxic to humans. These drugs can trigger a series of molecular changes in proteins involved in DNA damage repair, cell cycle, apoptosis, and necrosis, which are closely linked to cell sensitivity to radiation. These key proteins are the focus of research as potential targets of radiotherapy-sensitizing drugs (190). PARP inhibitors are rapidly emerging as effective anticancer drugs. Currently available second- and third-generation PARP inhibitors are efficient because of enhanced radiotherapy sensitivity, improved selectivity, and reduced toxicity (191–193). Overall, PARP1 inhibitors have potential clinical use as effective radiotherapy sensitizers in the near future.

## Author contributions

YZ: Writing – original draft, Writing – review & editing. LL: Writing – original draft, Writing – review & editing. ZL: Writing – original draft, Writing – review & editing. YH: Writing – original draft, Writing – review & editing. MJ: Writing – review & editing. BZ: Conceptualization, Supervision, Writing – review & editing. YX: Conceptualization, Supervision, Writing – review & editing.
